# Supplementary material for: Social motility of biofilm-like microcolonies in a gliding bacterium
Source: Nat Commun. 2021 Sep 29;12:5700. doi: 10.1038/s41467-021-25408-7 (PMC8481357; doi:10.1038/s41467-021-25408-7)
Supplement: Supplementary file 1 — Supplementary Information [file 41467_2021_25408_MOESM1_ESM.pdf]

Supplementary information

Social motility of biofilm-like microcolonies in a gliding bacterium

Chao Li[1†], Amanda Hurley[2,3†], Wei Hu[4], Jay W. Warrick[5], Gabriel L. Lozano[2,6], Jose M. Ayuso[1,5,7], Wenxiao Pan[4], Jo Handelsman[2,3] & David J. Beebe[1,5,8*]

[1]Carbone Cancer Center, University of Wisconsin-Madison, Madison, WI 53705, USA. [2]Wisconsin Institute for Discovery, University of Wisconsin-Madison, Madison, WI, 53715, USA. [3]Department of Plant Pathology, University of Wisconsin-Madison, Madison, WI, 53706, USA. [4]Department of Mechanical Engineering, University of Wisconsin-Madison, Madison, WI 53706, USA. [5]Department of Biomedical Engineering, University of Wisconsin-Madison, Madison, WI 53705, USA. [6]Divisions of Infectious Diseases and Gastroenterology, Boston Children's Hospital and Harvard Medical School, Boston, MA 02115, USA. [7]Morgridge Institute for Research, Madison, WI 53715, USA. [8]Department of Pathology and Laboratory Medicine, University of Wisconsin-Madison, Madison, WI, 53705, USA. [†]These authors contributed equally. [*]Correspondence should be addressed to D.J.B. (djbeebe@wisc.edu).

Supplementary Table 1 Strains used in this study.

Strain	Genotype	Source or reference
UW101 (ATCC17061)	Wild-type	1
CJ1922	*rpsL2 ΔsprB*	2
CJ1984	*rpsL2 ΔremA*	2,3
CJ1985	*rpsL2 ΔsprBremA*	3
CJ2005	CJ1984 + pRR39 (RemA inserted into pCP23)	3
fjoh_0352	Tn::*fjoh_0352*	This study
gldD	Tn::*fjoh_1540*	This study
lepA	Tn::*fjoh_0786*	This study

Supplementary Table 2 Primers used in constructing pSAM_*Fjoh2* vector.

Name	Sequence
ermF5_*Xho*I	CACCCTCGAGAACAGTGCTTTTATCTACTCCG
ermF3_*Xba*I	TCTAGACTACGAAGGATGAAATTTTTCAGGG
*fjoh_1433_Bam*HI	CACCGGATCCATCAAGATTCAGAAACTTCATT

*fjoh_1433*_TransRev	AAATTCCTTTTTTTCCATCTTATTTGTCTCCTCAAT
*fjoh_1433*_TransFor	ATTGAGGAGACAAATAAGATGGAAAAAAAGGAATTT
Transpo_Rev	TGCGGCCGCTTATTCAACATAGTTCCCTTC

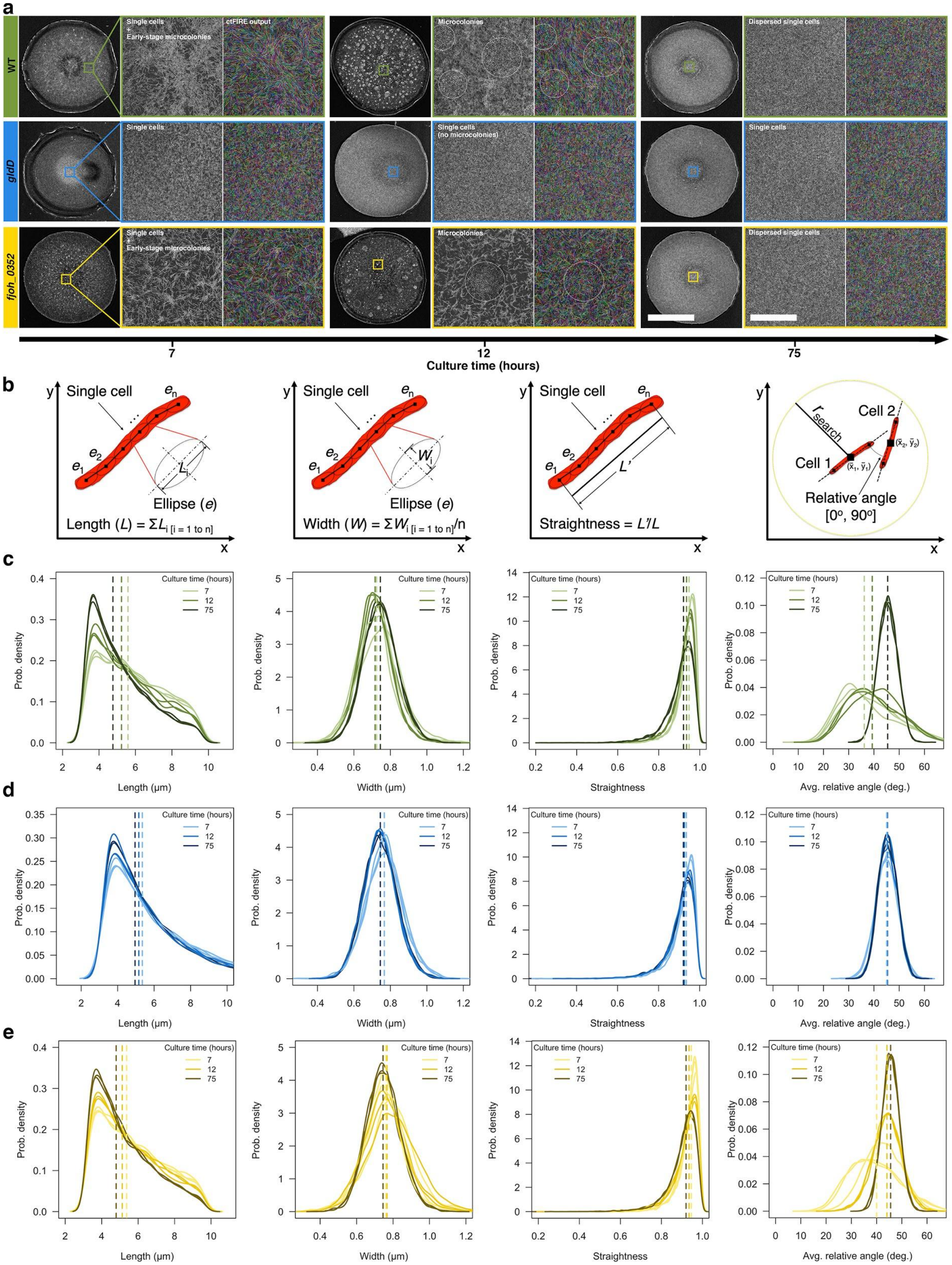

Supplementary Fig. 1 Evolution of single-cell organization on the surface and in microcolonies over time. **a**, Bright-field microscopic images at different culture times (7 hr, 12 hr, and 75 hr after starting culture). In

each condition and time point, the 6× microdrop image is displayed on the left, the corresponding 60× image is in the middle [range of interest (ROI) shown as the square box on the 6× image], and the 60× ctFIRE output image is shown with the multicolored rendering on the right. Typical microcolonies at 12 hr are highlighted with white dotted-line circles. Scale bars, 1 mm for the 6× microdrop images and 100 μm for the 60× images. The experiments were performed three times for the representative result. **b**, Schematics show the algorithms of length, width, straightness, and the relative angle. Ellipses were generated in ctFIRE to fit and fill the objects of interest by scanning each image. The length of a bacterium (*L*) is resolved as the sum of the length of each ellipse (L_i). The width of a bacterium (*W*) is resolved as the arithmetic mean of the width of each ellipse (W_i). The straightness of a bacterium is resolved as the Euclidean distance between the centers of the first and the end ellipses (*L*') divided by the cell length (*L*). The relative angle between two bacteria is defined as the angle between the axes that pass through each bacterium by connecting the centers of the first and the last ellipses associated with each cell. The location of a bacterium is revolved as the arithmetic means of the x- and y-coordinates of the fitting ellipses. An ROI with a search radius r_{search} = 9.8 μm (the yellow circle) was set to find the neighbor cells (e.g. Cell 2) of the bacterium of interest (e.g. Cell 1). The relative angles between the bacterium of interest and its neighbor cells were calculated into a weighted mean. The range of relative angle is from 0° to 90°, with 45° indicating a random alignment of the bacteria. **c**, Wild-type (WT) (green), **d**, *gldD* (blue), and **e**, *fjoh_0352* (yellow), histograms of each of the analyzed parameters in (**b**). The dashed lines show the pooled median of each time point for three biological replicates (except *gldD* at 12 hr which only had two). Over time, WT and *fjoh_0352* displayed decreased cell length and alignment, due to projection into the focal plane during the formation of microcolonies (thus more cells holding an angle relative to the surface) during early growth phase (< 15 hr) and the dispersal of microcolonies (with most of the cells detached from the surface and suspending randomly in the media) in the late stage (> 15 hr). *gldD* remained in a random arrangement through its entire growth. All strains maintained a relatively constant width but became slightly curly over time with a decreased straightness. Source data are provided as a Source Data file.

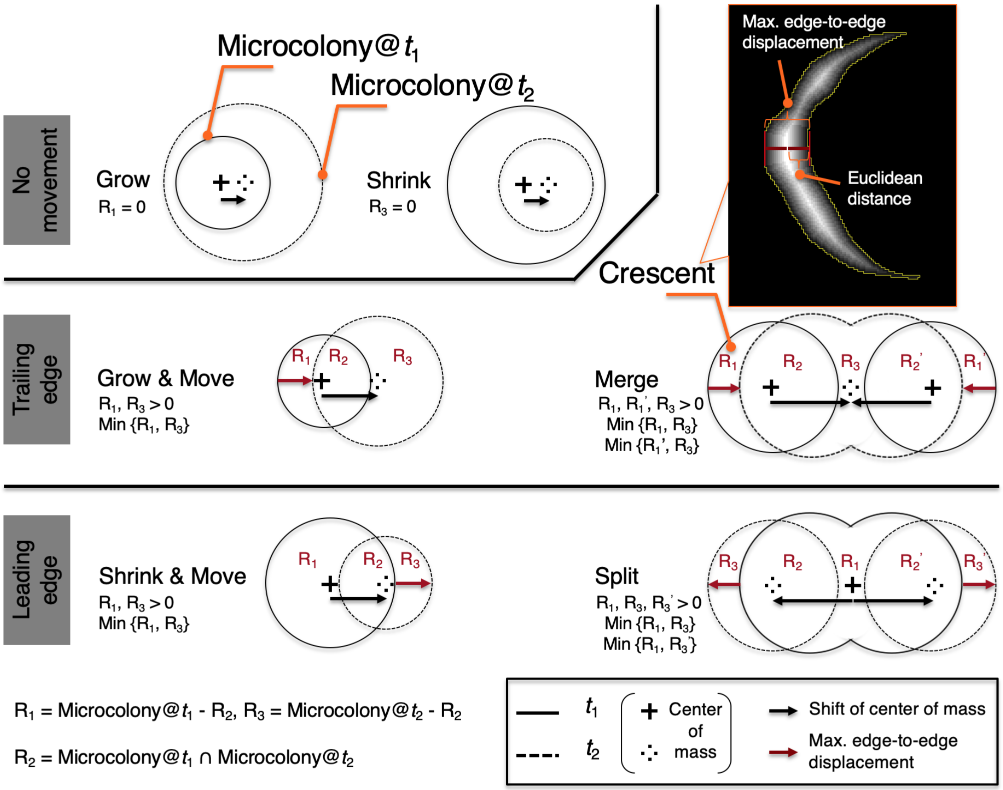

Supplementary Fig. 2 Schematic of the particle tracking algorithm. During movement, microcolonies may grow, shrink, merge, or split. These events can cause a sudden increase in the location of the center-of-mass (i.e. a positive error on microcolony motility). In contrast, the edge-to-edge displacement at either the trailing edge (for grow & move or merge) or the leading edge (for shrink & move, or split) is robust to these events but only provides information on motility, rather than precise position. The edge-to-edge displacement was extracted from the crescent obtained by superimposing two adjacent frames at t_1 (the solid line circle) and t_2 (the dashed line circle) in the time lapse. The area of the crescent at the trailing edge is designated R_1, at the leading edge designated R_3. The intersection between microcolony@t_1 and microcolony@t_2 is designed R_2 (i.e. R_2 = microcolony@t_1 ∩ microcolony@t_2), which resolves R_1 = microcolony@t_1 - R_2, and R_3 = microcolony@t_2 - R_2. The trailing edge ($R_1 < R_3$) or the leading edge ($R_1 > R_3$) can be distinguished by comparing R_1 and R_3. An Euclidean distance method was used to find the maximum edge-to-edge displacement along the normal at a point on the edge from the crescent showing a smaller area (i.e. Min {R_1, R_3}), which is then used for the following calculation of microcolony motility. The same principle applies to R_1', R_2', and R_3'. Center-of-mass was used to generate spider plots of the track displacement that show the microcolony positions over time. The motility measured by edge-to-edge displacement was used to remove spurious movements from the spider plots.

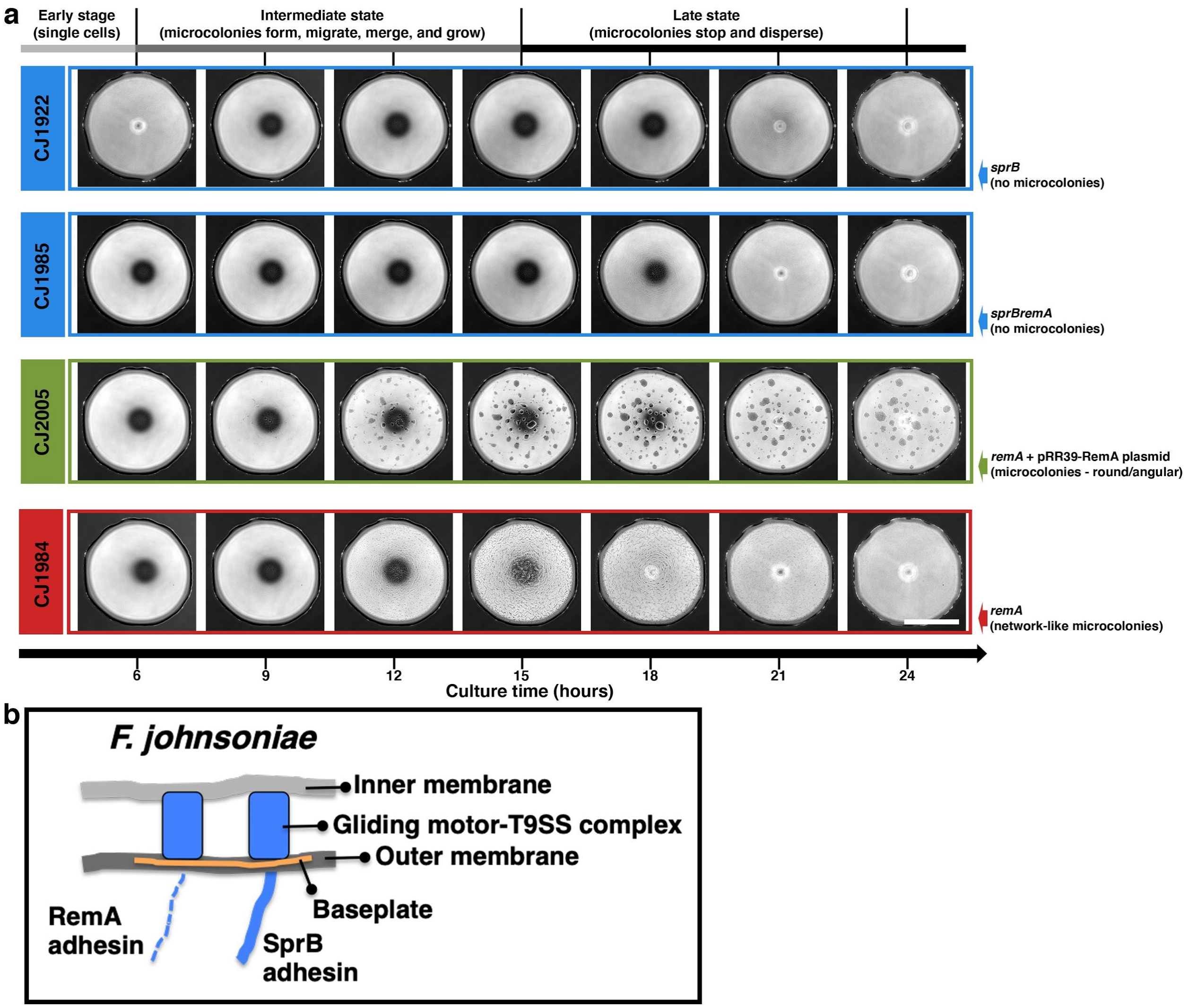

a
Early stage
(single cells)
Intermediate state
(microcolonies form, migrate, merge, and grow)
Late state
(microcolonies stop and disperse)
CJ1922
sprB
(no microcolonies)
CJ1985
sprBremA
(no microcolonies)
CJ2005
remA + pRR39-RemA plasmid
(microcolonies - round/angular)
CJ1984
remA
(network-like microcolonies)
6
9
12
15
18
21
24
Culture time (hours)
b
F. johnsoniae
Inner membrane
Gliding motor-T9SS complex
Outer membrane
Baseplate
RemA
adhesin
SprB
adhesin

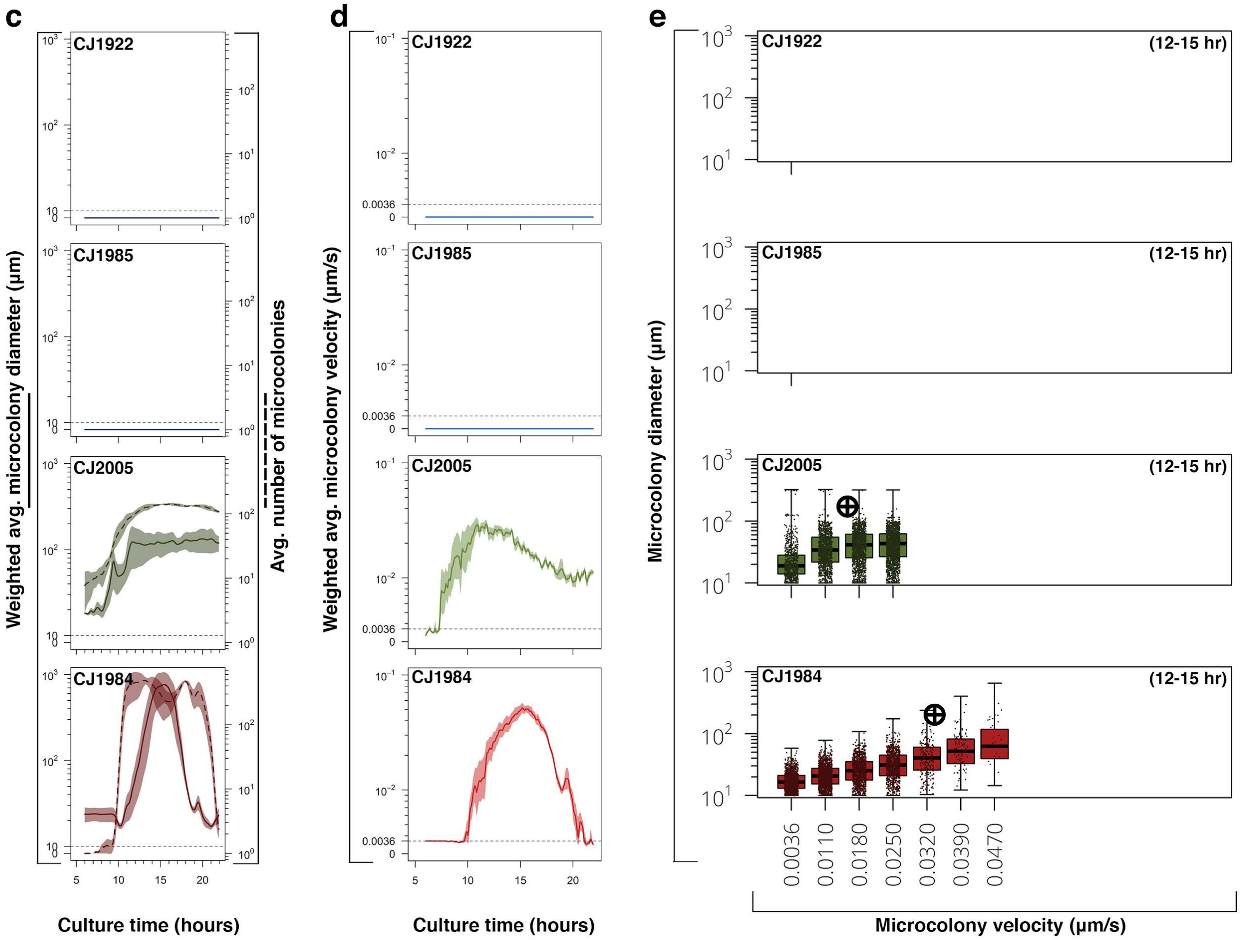

Supplementary Fig. 3 Growth dynamics and motility analysis of *sprB* and *remA* mutant microcolonies. **a**, Comparison matrix with bright-field microscopic images of the mutants. Images of *F. johnsoniae* wild-type (WT) are shown in Fig. 2b. Scale bar, 1 mm. The experiments were performed three times for the representative result. **b**, Schematic showing the gliding apparatus of *F. johnsoniae* and the genetic defects on each mutant, i.e. CJ1922 (*sprB*), CJ1985 (*sprBremA*), CJ2005 (*remA* + pRR39-RemA plasmid), and CJ1984 (*remA*). **c**, Weighted average microcolony diameter (solid line) and average number of microcolonies (dashed line) versus culture time (step, 5 min). **d**, Weighted average microcolony velocity versus culture time (step, 5 min). Estimated microcolony volumes were used as weights for all weighted average calculations. The standard error is represented by the envelope on the plots in (**c**) and (**d**). **e**, Strip plots showing the distribution of microcolony diameter versus microcolony velocity during growth (6-9, 9-12, 12-15, and 15+ hr). Data points were pooled from three biological replicates. Crosses indicate medians. Box edges and center represent the 25th, 75th, and 50th percentile while whiskers represent 1.5×IQR (interquartile range) or the minima and maxima (whichever is less). Source data are provided as a Source Data file.

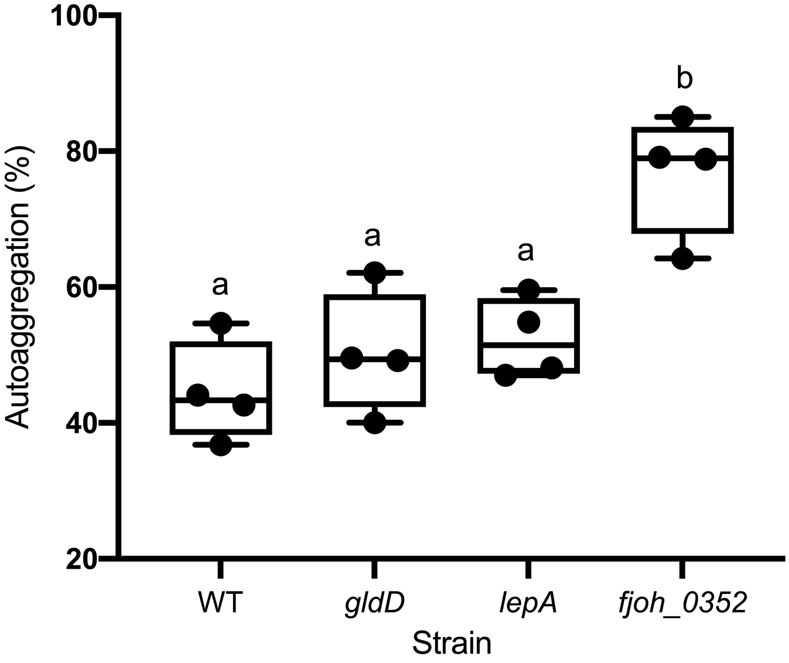

Supplementary Fig. 4 Autoaggregation of *F. johnsoniae* mutants. Percent of bacterial culture, measured by optical density at the wavelength of 600 nm (OD_{600}), that settles over a 24-hr period[4] using the following equation: 100 - (average final OD_{600}/start OD_{600}) × 100%. Multiple transposon mutants were included in the analysis to control for the production of erythromycin resistance conferred by the transposon. The ability of *fjoh_0352* to autoaggregate was enhanced compared to wild-type (WT) and other transposon mutants, including *gldD.* Boxplots display the median and 10th-90th quartiles of four biological replicates, each the average of two technical replicates, in a single experiment. Different lowercase letters indicate groups differ significantly according to a non-parametric, two-tailed t-test (P = 0.0286). Source data are provided as a Source Data file.

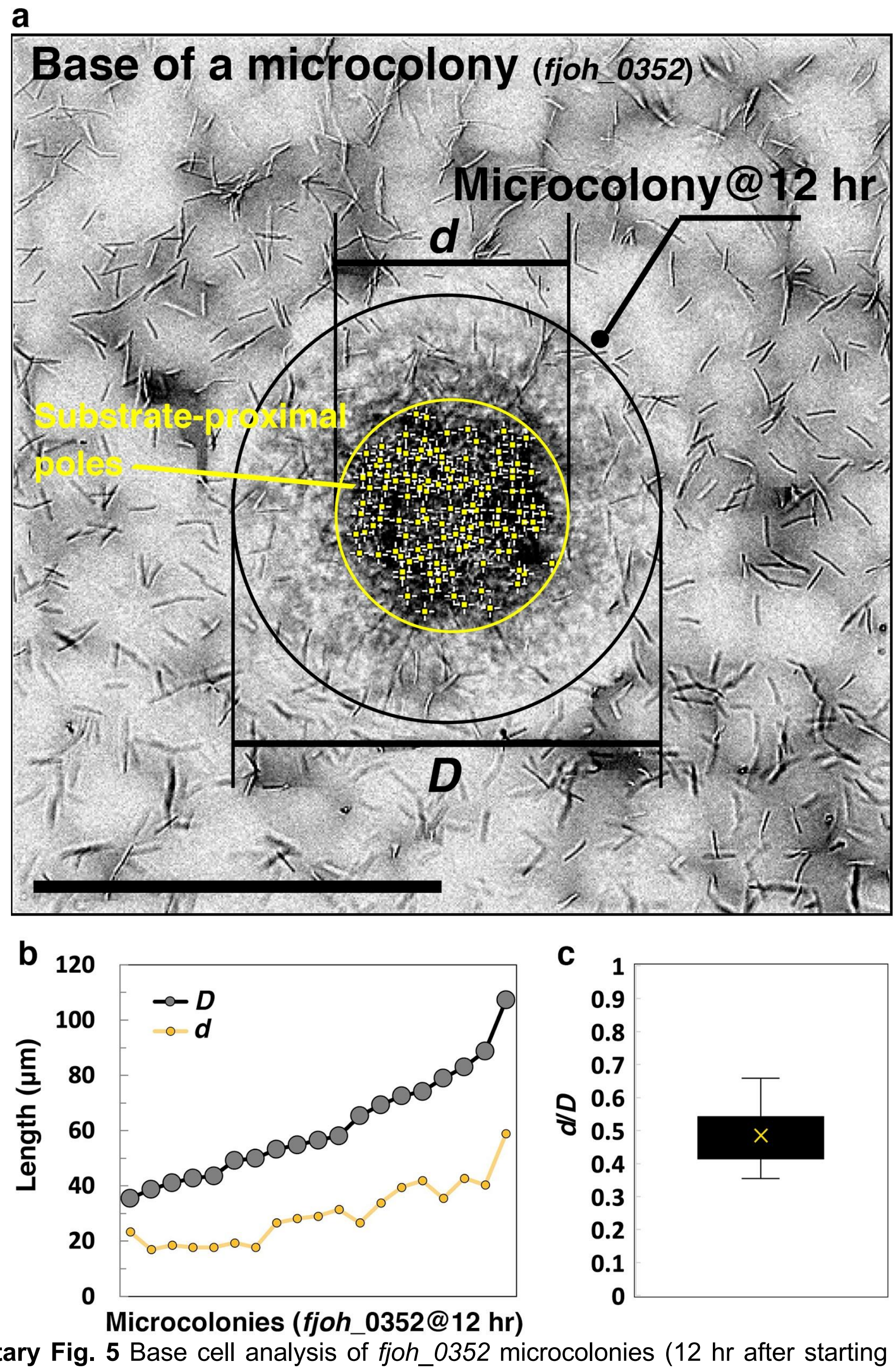

Supplementary Fig. 5 Base cell analysis of *fjoh_0352* microcolonies (12 hr after starting culture). **a**, A "Find Maxima" result in Fiji ImageJ that captures the base cells at the substrate-proximal side of a microcolony. Each yellow cross represents a cell pole, which in total gives the number of base cells of a microcolony. Scale bar, 100 µm. The experiments were performed three times for the representative result. **b**, Comparison between the microcolony diameter (*D*) and the base diameter (*d*). **c**, Distribution of *d*/*D*. Box edges and center (i.e. the cross) represent the 25th, 75th, and 50th percentile (average *d*/*D* = 0.48) while whiskers represent the minima and maxima. Source data are provided as a Source Data file.

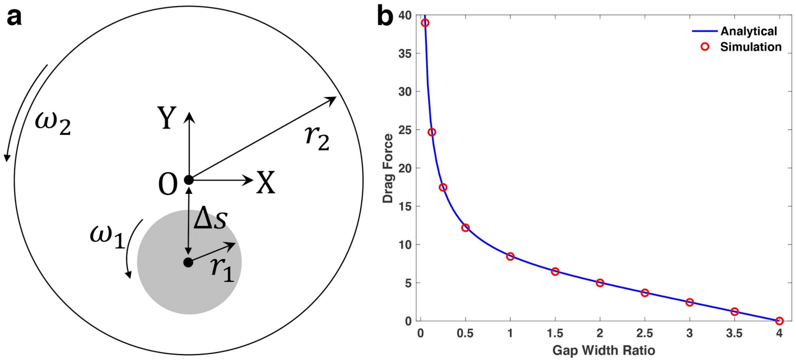

Supplementary Fig. 6 Benchmark test of the numerical method. **a.** Setup of Wannier flow problem. **b.** Drag force (Unit: $\times 10^{-15}$ N) per micrometer length exerted on the inner cylinder in Wannier flow as a function of ratio of the minimum gap width to the inner cylinder's radius, i.e., $\frac{(r_2 - r_1 - \Delta s)}{r_1}$, where the simulation results are compared with the analytical solution[5]. Here, $r_1 = \frac{\pi}{10} \times 10^{-3} m$, $r_2 = \frac{\pi}{2} \times 10^{-3} m$, $\omega_1 = \frac{10}{\pi} \times 10^{-3} rad/s$, and $\omega_2 = \frac{1}{\pi} \times 10^{-3} rad/s$.

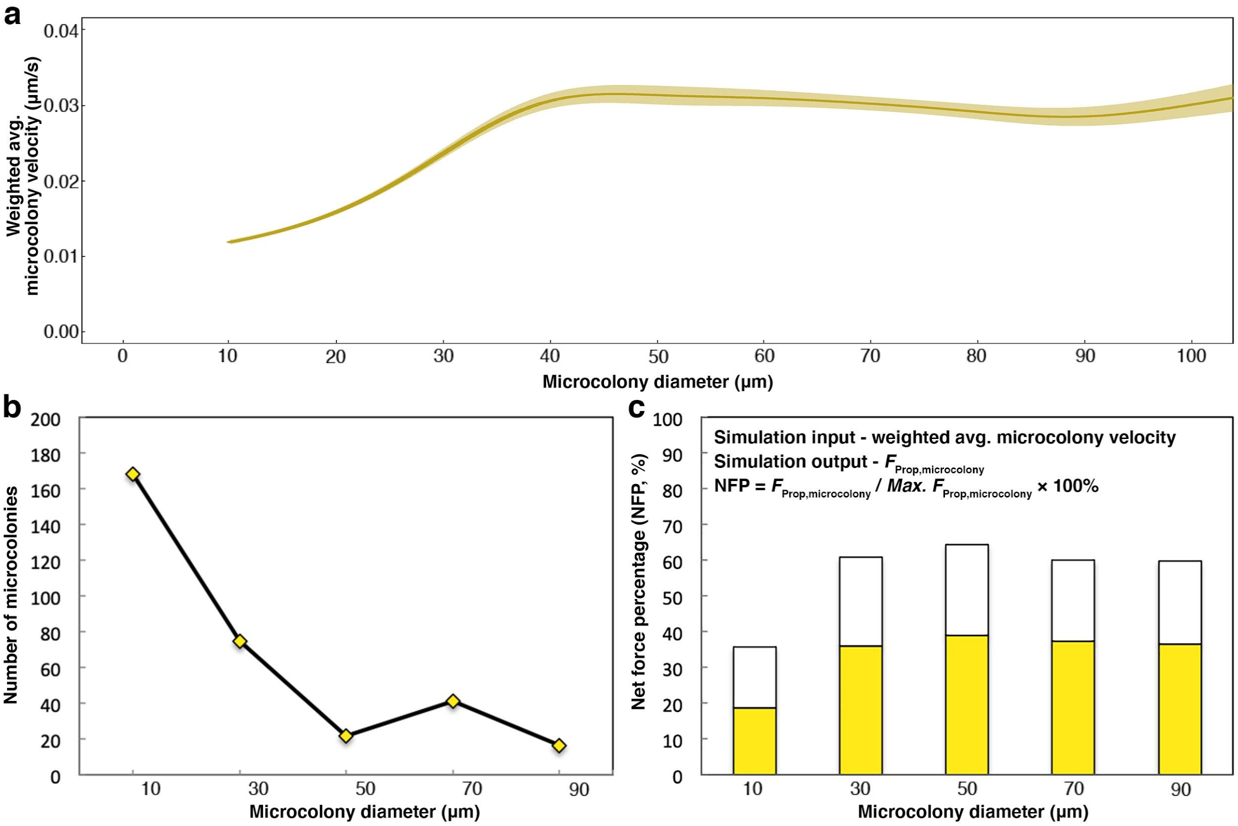

Supplementary Fig. 7 Velocity, density, and net force percentage (NFP) of microcolonies produced by *fjoh_0352* 11.5-12.5 hr after starting culture. **a**, Weighted average microcolony velocity as a function of microcolony diameter. Estimated microcolony volumes were used as weights for all weighted average calculations. The standard error is represented by the envelope on the plots. **b**, Number of microcolonies in an under-oil microdrop for a given microcolony diameter. The experiments were performed three times for the representative result. **c**, NFP corresponding to different microcolony diameters. Note that if the maximum velocity (0.075 µm/s) is used as input from the most motile subgroup of microcolonies, the NFP

can reach as high as 91%. Error bars, mean (the yellow bar) ± s.d. (the white bar). The simulated results were obtained based on the averaged input data from three biological replicates. Source data are provided as a Source Data file.

Supplementary references

1. McBride, M. J. & Braun, T. F. GldI is a lipoprotein that is required for *Flavobacterium johnsoniae* gliding motility and chitin utilization. *J. Bacteriol.* **186**, 2295–2302 (2004).
2. Rhodes, R. G., Pucker, H. G. & McBride, M. J. Development and use of a gene deletion strategy for *Flavobacterium johnsoniae* to identify the redundant gliding motility genes *remF*, *remG*, *remH*, and *remI*. *J. Bacteriol.* **193**, 2418–2428 (2011).
3. Shrivastava, A., Rhodes, R. G., Pochiraju, S., Nakane, D. & McBride, M. J. *Flavobacterium johnsoniae* RemA is a mobile cell surface lectin involved in gliding. *J. Bacteriol.* **194**, 3678–3688 (2012).
4. Sorroche, F. G., Spesia, M. B., Zorreguieta, A. & Giordano, W. A positive correlation between bacterial autoaggregation and biofilm formation in native *Sinorhizobium meliloti* isolates from Argentina. *Appl. Environ. Microbiol.* **78**, 4092–4101 (2012).
5. Wannier, G. H. A contribution to the hydrodynamics of lubrication. *Q. Appl. Math.* **8**, 1–32 (1950).
