# Supplementary material for: Social motility of biofilm-like microcolonies in a gliding bacterium
Source: Nat Commun. 2021 Sep 29;12:5700. doi: 10.1038/s41467-021-25408-7 (PMC8481357; doi:10.1038/s41467-021-25408-7)
Supplement: Supplementary file 3 — Description of Additional Supplementary Files [file 41467_2021_25408_MOESM3_ESM.pdf]

Description of Additional Supplementary Files

File Name: Supplementary Movie 1
Description: Growth dynamics of *F. johnsoniae* WT (6×, 6-24 hr time lapse, 3000× accelerated). At t = 0 hr, 2 μl *F. johnsoniae* WT was inoculated to each under-oil spot (2 mm in diameter) at an inoculum density of 10^7 CFU/ml. Growth was recorded by bright-field time lapse at 6× magnification from 6 to 24 hr. In the video, 1 s equals 50 min in real time.

File Name: Supplementary Movie 2
Description: EPS cores in the *F. johnsoniae fjoh_0352* microcolonies (12 hr after starting culture, ConA lectin staining, 60×, z-stack from bottom to top). Under-oil spots (2 mm in diameter) were inoculated with 2 μl of *F. johnsoniae fjoh_0352* at an inoculum density of 10^7 CFU/ml. At 12 hr post inoculation, the microcolonies were stained with ConA lectin (0.2 μl/spot at a final concentration of 0.005 mg/ml). The z-stack video was recorded at 60× magnification at 30 min after adding the ConA lectin. The split screens on the left and right show the bright-field channel and the (Ex/Em) 555/580 nm channel, respectively.

File Name: Supplementary Movie 3
Description: Growth dynamics of *F. johnsoniae gldD* (6×, 6-24 hr time lapse, 3000× accelerated). At t = 0 hr, under-oil spots (2 mm in diameter) were inoculated with 2 μl of *F. johnsoniae gldD* at an inoculum density of 10^7 CFU/ml. Growth was recorded by bright-field time lapse at 6× magnification from 6 to 24 hr. In the video, 1 s equals 50 min in real time.

File Name: Supplementary Movie 4
Description: Growth dynamics of *F. johnsoniae fjoh_0352* (6×, 6-24 hr time lapse, 3000× accelerated). At t = 0 hr, under-oil spots (2 mm in diameter) were inoculated with 2 μl of *F. johnsoniae fjoh_0352* at an inoculum density of 10^7 CFU/ml. Growth was recorded by bright-field time lapse at 6× magnification from 6 to 24 hr. In the video, 1 s equals 50 min in real time.

File Name: Supplementary Movie 5
Description: Microdrop of *F. johnsoniae* WT (-) CCCP (6×, 6-12 hr time lapse, 3000× accelerated). At t = 0 hr, under-oil spots (2 mm in diameter) were inoculated with 2 μl of *F. johnsoniae* WT at an inoculum density of 10^7 CFU/ml. Growth was recorded by bright-field time lapse at 6× magnification from 6 to 12 hr. In the video, 1 s equals 50 min in real time.

File Name: Supplementary Movie 6
Description: Microdrop of *F. johnsoniae* WT (+) CCCP (6×, 15-22 hr time lapse, 3000× accelerated). At 12 hr post inoculation, 10 μM of carbonyl cyanide m-chlorophenylhydrazine (CCCP) was added to the microdrops and the video was recorded by bright-field time lapse at 6× magnification from 15 to 22 hr. In the video, 1 s equals 50 min in real time.

File Name: Supplementary Movie 7
Description: Microdrop of *F. johnsoniae fjoh_0352* (-) CCCP (6×, 6-12 hr time lapse, 3000× accelerated). At t = 0 hr, under-oil spots (2 mm in diameter) were inoculated with 2 μl of *F. johnsoniae fjoh_0352* at an inoculum density of 10^7 CFU/ml. Growth was recorded by bright-field time lapse at 6× magnification from 6 to 12 hr. In the video, 1 s equals 50 min in real time.

File Name: Supplementary Movie 8
Description: Microdrop of *F. johnsoniae fjoh_0352* (+) CCCP (6×, 15-22 hr time lapse, 3000× accelerated). At 12 hr post inoculation, 10 μM of carbonyl cyanide m-chlorophenylhydrazine (CCCP) was added to the microdrops and the video was recorded by bright-field time lapse at 6× magnification from 15 to 22 hr. In the video, 1 s equals 50 min in real time.

File Name: Supplementary Movie 9

Description: Pinwheel of *F. johnsoniae* WT cells during the early-stage microcolony formation (7 hr post inoculation, 60×, 30 s, 2.5× accelerated). At t = 0 hr, under-oil spots (2 mm in diameter) were inoculated with 2 μl of *F. johnsoniae* WT at an inoculum density of 10^7 CFU/ml. Growth was recorded by bright-field time lapse at 60× magnification. Pinwheeling motion was captured at 7 hr post inoculation. The focal plane was set about 5 μm above the glass surface in a microdrop. In the video, 1 s equals 2.5 min in real time.

File Name: Supplementary Movie 10
Description: Merging of *F. johnsoniae fjoh_0352* microcolonies (12 hr after starting culture, 60×, 30 s, 2.5× accelerated). At t = 0 hr, under-oil spots (2 mm in diameter) were inoculated with 2 μl of *F. johnsoniae fjoh_0352* at an inoculum density of 10^7 CFU/ml. Growth was recorded by bright-field time lapse at 60× magnification. The merging of microcolonies was captured at 12 hr post inoculation. The focal plane was set on the glass surface in a microdrop. In the video, 1 s equals 2.5 min in real time.
