# Supplementary material for: Social motility of biofilm-like microcolonies in a gliding bacterium
Source: Nat Commun. 2021 Sep 29;12:5700. doi: 10.1038/s41467-021-25408-7 (PMC8481357; doi:10.1038/s41467-021-25408-7)
Supplement: Supplementary file 14 — Reporting Summary [file 41467_2021_25408_MOESM14_ESM.pdf]

nature research

Corresponding author(s): David J. Beebe

Last updated by author(s): 05/08/2021

Reporting Summary

Nature Research wishes to improve the reproducibility of the work that we publish. This form provides structure for consistency and transparency in reporting. For further information on Nature Research policies, see our Editorial Policies and the Editorial Policy Checklist.

Statistics

For all statistical analyses, confirm that the following items are present in the figure legend, table legend, main text, or Methods section.

n/a	Confirmed	
☐	☒	The exact sample size (*n*) for each experimental group/condition, given as a discrete number and unit of measurement
☐	☒	A statement on whether measurements were taken from distinct samples or whether the same sample was measured repeatedly
☐	☒	The statistical test(s) used AND whether they are one- or two-sided<br>*Only common tests should be described solely by name; describe more complex techniques in the Methods section.*
☐	☒	A description of all covariates tested
☐	☒	A description of any assumptions or corrections, such as tests of normality and adjustment for multiple comparisons
☐	☒	A full description of the statistical parameters including central tendency (e.g. means) or other basic estimates (e.g. regression coefficient) AND variation (e.g. standard deviation) or associated estimates of uncertainty (e.g. confidence intervals)
☐	☒	For null hypothesis testing, the test statistic (e.g. *F*, *t*, *r*) with confidence intervals, effect sizes, degrees of freedom and *P* value noted<br>*Give P values as exact values whenever suitable.*
☒	☐	For Bayesian analysis, information on the choice of priors and Markov chain Monte Carlo settings
☒	☐	For hierarchical and complex designs, identification of the appropriate level for tests and full reporting of outcomes
☒	☐	Estimates of effect sizes (e.g. Cohen's *d*, Pearson's *r*), indicating how they were calculated

Our web collection on statistics for biologists contains articles on many of the points above.

Software and code

Policy information about availability of computer code

Data collection	NIS-Elements AR (from Nikon) was used to collect images/time lapse videos.
Data analysis	Fiji ImageJ was used to quantify the base cells and the effect of CCCP. JEX (an open-source image analysis software that uses well-established libraries from ImageJ) was used to build a custom particle tracking algorithm for analyzing the time lapse videos. CT-FIRE for Individual Fiber Extraction (Current Version: V2.0 Beta) was used to perform the single-cell organization analysis. GMLS method was used to carry out the computational simulation for quantifying the level of synchronization of base cells in microcolony motility.

For manuscripts utilizing custom algorithms or software that are central to the research but not yet described in published literature, software must be made available to editors and reviewers. We strongly encourage code deposition in a community repository (e.g. GitHub). See the Nature Research guidelines for submitting code & software for further information.

Data

Policy information about availability of data

All manuscripts must include a data availability statement. This statement should provide the following information, where applicable:

- Accession codes, unique identifiers, or web links for publicly available datasets
- A list of figures that have associated raw data
- A description of any restrictions on data availability

Data availability
All data are available from the corresponding authors upon reasonable request. Source data are provided with this paper.

Field-specific reporting

Please select the one below that is the best fit for your research. If you are not sure, read the appropriate sections before making your selection.

☒ Life sciences ☐ Behavioural & social sciences ☐ Ecological, evolutionary & environmental sciences

For a reference copy of the document with all sections, see nature.com/documents/nr-reporting-summary-flat.pdf

Life sciences study design

All studies must disclose on these points even when the disclosure is negative.

Sample size	This work reports the discovery of a new mode of social biofilm motility of bacteria. No sample size was previously calculated. We performed the UOMS culture with all of the bacterial strains in this study for at least three (4 to 7) independent biological replicates.
Data exclusions	We excluded a set of data from only one replicate because an abnormally low growth dynamics was found in that specific test (see below).
Replication	All attempts at replication were successful except for one and we figured out it was caused by an operation error in the pre-culture of the bacteria before that test.
Randomization	We discovered the social biofilm motility first in an experiment with the wide-type. We selected the mutants by mainly targeting on the gliding apparatus and surface property of the bacteria.
Blinding	In this study, data collection, analysis, and computational simulation were done through a collaboration of three independent labs. All of the data if no error in operation was found were pooled together to avoid bias in analysis.

Reporting for specific materials, systems and methods

We require information from authors about some types of materials, experimental systems and methods used in many studies. Here, indicate whether each material, system or method listed is relevant to your study. If you are not sure if a list item applies to your research, read the appropriate section before selecting a response.

Materials & experimental systems

n/a	Involved in the study	
☒	☐	Antibodies
☒	☐	Eukaryotic cell lines
☒	☐	Palaeontology and archaeology
☒	☐	Animals and other organisms
☒	☐	Human research participants
☒	☐	Clinical data
☒	☐	Dual use research of concern

Methods

n/a	Involved in the study	
☒	☐	ChIP-seq
☒	☐	Flow cytometry
☒	☐	MRI-based neuroimaging
